# Value of Carbohydrate Antigen 19-9 in Predicting Response and Therapy Control in Patients with Metastatic Pancreatic Cancer Undergoing First-Line Therapy

**DOI:** 10.3389/fonc.2013.00155

**Published:** 2013-06-14

**Authors:** Uwe Pelzer, Andreas Hilbig, Marianne Sinn, Jens Stieler, Marcus Bahra, Bernd Dörken, Hanno Riess

**Affiliations:** ^1^Department of Hematology/Oncology, Comprehensive Cancer Center, Universitätsmedizin Berlin – Charité, Berlin, Germany; ^2^Department of Surgery, Universitätsmedizin Berlin – Charité, Berlin, Germany

**Keywords:** pancreatic neoplasms, CA 19-9, response, therapy control, prediction

## Abstract

**Background:** Serum carbohydrate antigen 19-9 (CA 19-9) has been shown to be a sensitive and specific serum marker for pancreatic cancer. Little has been published about correlations between baseline CA 19-9 level or changes to CA 19-9 level and median overall survival (mOS). Its impact on monitoring treatment efficacy remains under discussion, however.

**Methods:** CA 19-9 serum level was measured in 181 consecutive patients with advanced pancreatic cancer (APC) being treated with gemcitabine-based first-line chemotherapy. We separated the patients into several groups depending on baseline CA 19-9 levels and the CA 19-9 response after 6–8 weeks of treatment. Evaluations were made using SPSS 19.9.

**Results:** Median baseline CA 19-9 level was 1,493 U/ml (range 40–1,043,301). Patients with baseline CA 19-9 ≤1,000 U/ml had a mOS of 14.9 months (95% CI: 11.36:18.44), whereas patients with CA 19-9>1,000 U/ml had a mOS of 7.4 months [(95% CI: 5.93:8.87) *p* < 0.001, HR 2.12]. With regard to the change in CA 19-9 after 6–8 weeks of treatment: patients with increased CA 19-9 levels had a mOS of 8.1 months, those with stabilized CA 19-9 levels 11.6 months, and those with decreased CA 19-9 levels 11.1 months (*p* < 0.019).

**Conclusion:** CA 19-9 levels can separate patients with differing mortality risks at baseline. Patients with stabilization or high response of CA 19-9 after 6–8 weeks of treatment had no significant differences in survival rates, whereas patients with increased CA 19-9 had significantly lower survival rates, indicating an early treatment failure.

## Introduction

Despite global advances in oncology, pancreatic cancer is still a devastating disease with poor prognosis. It remains one of the leading causes of cancer-related deaths worldwide, reflected by an incidence of 277,668 new cases and almost the same mortality rate (266,029 cases) per year (GLOBOCAN, 2008). Due to early disease symptoms being missed, only up to 20% of patients can have their cancer resected with curative intent, however, probably due to early lymphatic spread or micrometastasis, the 5-year overall survival rate of resected patients is only 15–22% (Neoptolemos et al., 2004; Oettle et al., 2007; Gillen et al., 2010) in spite of adjuvant treatment.

The majority of patients presenting with advanced disease have a 5-year survival rate below 5%. The 1-year survival rate ranges between 18 and 40% (Burris et al., 1997; Moore et al., 2005; Herrmann et al., 2007; Conroy et al., 2011), depending on treatment design. Current strategy is that patients with better performance status (0–1) receive combined chemotherapy using a gemcitabine-based regimen (Louvet et al., 2005; Heinemann et al., 2008; Pelzer, 2008a; Cunningham et al., 2009) or a gemcitabine-free combination (Conroy et al., 2011). The majority of patients with a moderate performance status should be treated with gemcitabine monotherapy, or in selected cases in combination-therapy with erlotinib (Moore et al., 2005). The overall benefit of a second-line therapy was proven in a phase III setting (Pelzer et al., 2011), additionally many phase II trials have demonstrated moderate efficacy of several treatment options (Pelzer, 2008b). For individualized therapy it is important that refractory disease is detected as early as possible to enable further treatment strategies to be offered and thus increase the benefit of adopted second-line treatment and hopefully prevent side effects due to ineffective therapy. Computed tomography or magnetic resonance imaging are still the reference methods for evaluating the response to chemotherapy treatment, but are an expensive and not always reliable assessment method (Ishii et al., 2005).

Many different biomarkers have been studied in the last decade in the hope of finding a simpler evaluation tool for physicians. Firstly in order to detect more patients at an early tumor stage, and secondly in order to determine the efficacy of tumor treatment so that current treatment strategies can be modified. Serum carbohydrate antigen 19-9 (CA 19-9) – first described by Koprowski et al. (1979) – has been shown to be a sensitive and specific serum marker for pancreatic cancer (Ballehaninna and Chamberlain, 2011). About 10% of the general population are lacking the Lewis blood group antigen and thus are not able to express the carbohydrate antigen (Lamerz, 1999; Duffy et al., 2010). We evaluated this proven biomarker in our patients to investigate the impact of CA 19-9 serum levels on survival.

## Materials and Methods

For this retrospective analysis we included patients with histologically confirmed advanced pancreatic cancer (APC) from our outpatient department who were treated with gemcitabine or gemcitabine-based first-line chemotherapy in our department between 1998 and 2005. Blood samples had been taken at treatment begin and every 6–8 weeks after and it was possible to follow up for overall survival.

Patients with a second malignancy or whose bilirubin levels were higher than the upper normal limit were excluded to avoid elevated CA 19-9 serum level due to other causes.

Patients were initially separated into two groups depending on baseline CA 19-9 serum level (≤1,000 vs.>1,000 U/ml) to detect the assumed prognostic impact of the CA 19-9 serum base line value. Furthermore, to investigate the predictive impact of the change of the CA 19-9 serum level within the first 6–8 weeks, we separated the patients into three groups according to its CA 19-9 serum level response within 6–8 weeks of initial treatment (>50% decrease in CA 19-9 serum value vs. 50% decrease to 20% increase vs.>20% increase in CA 19-9 serum value). Median overall survival (mOS) was defined as the duration between start of treatment and patient death from any cause. CA 19-9 serum levels were determined via electrochemiluminescence immunoassay (Elecsys 2010, Roche Diagnostics, upper normal limit of 37 U/ml).

Categorical variables were described by absolute and relative frequencies; age and CA 19-9 serum level were reported as median, ranges, arithmetic averages, and percentages from baseline. The overall survival was reported as median with 95% confidence interval, discrimination between the subgroups was done using the log-rank test and the cox proportional-hazard model. The graphic survival presentation was done using the Kaplan–Meier estimation (IBM SPSS 19.0).

## Results

**Figure 1:**
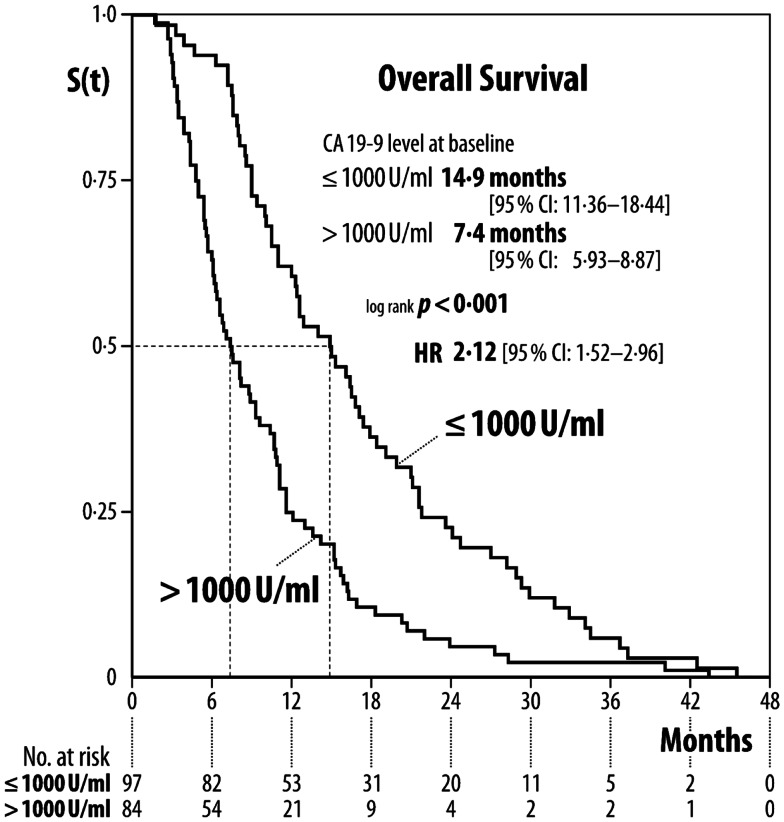
**Median overall survival according to CA 19-9 serum level baseline level**.

Two hundred fifty patients were screened and 181 patients were included in our analysis (Table 1). One hundred fifty (82.9%) patients had elevated baseline carbohydrate antigen 19-9 level above the upper limit of normal (>37 U/ml) and were selected for further specific analysis. Four (12.9%) out of 31 patients had elevated CA 19-9 serum level 6–8 weeks after treatment start in spite of normal baseline CA 19-9 serum level. The median serum level of CA 19-9 in 150 patients was 1,493 (40–1,043,301) U/ml at treatment begin; median age was 64 (range: 33–101) years. mOS of the 150 patients with upfront elevated CA19-9 level was 10.5 months (95% CI: 9.10–11.90). Patients with baseline CA19-9 ≤1,000 U/ml (*n* = 66) had a mOS of 14.9 (95% CI: 11.36–18.44), whereas patients with baseline CA 19-9 serum level > 1,000 U/ml (*n* = 84) had a poorer mOS of 7.4 (95% CI: 5.93–8.87) [log-rank: *p* < 0.001; HR 2.12 (95% CI: 1.52–2.96)] (Figures 1 and 3).

**Table 1:** **Patients’ baseline characteristics**.

Characteristic	
Included patients with pos. CA 19-9	181
Patients with CA 19-9>37 U/ml at baseline	150
**AGE (YEARS)**	
Median	64
Range	33–101
**GENDER (*n*)**	
Female	66 (44%)
Male	84 (66%)
**STAGE (*n*)**	
*M*_0_	31 (20.7%)
*M*_1_	119 (79.3%)
**BASELINE CA 19-9 (U/ml)**	
Median	1,493
Arithmetic mean	23,488
Range	40–1,043,301
**FIRST-LINE TREATMENT (*n*)**	
Gem	79 (52.7%)
Gem/folinic acid/5-FU	71 (47.3%)
**OVERALL SURVIVAL (MONTHS)**	
Median	10.5
95% CI	9.1–11.9

**Figure 2:**
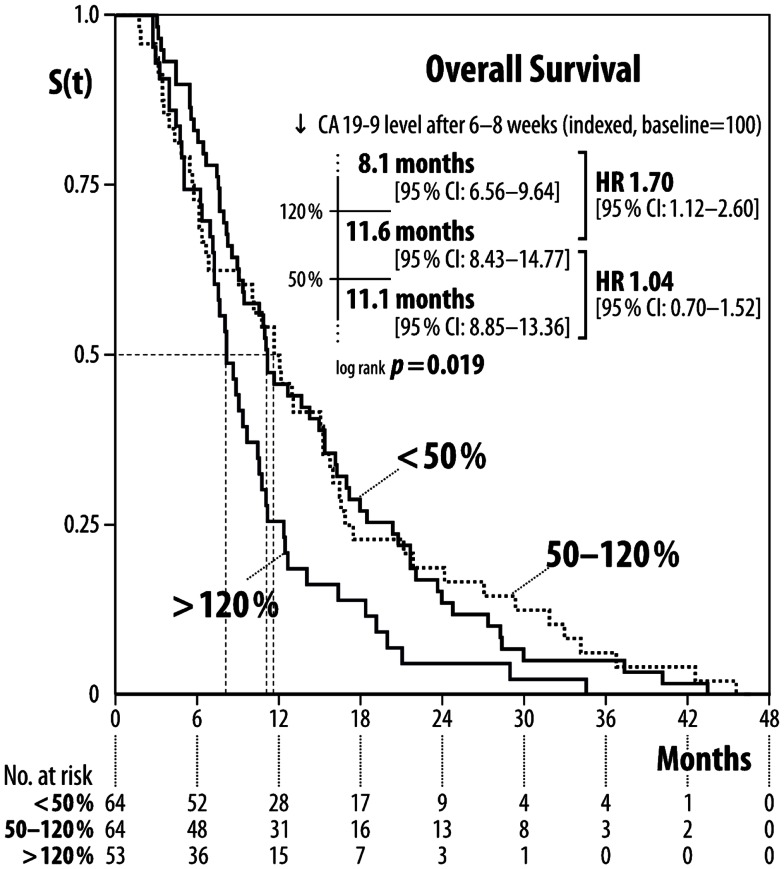
**Median overall survival according to changes in CA 19-9 level after 6–8 weeks of treatment**.

**Figure 3:**
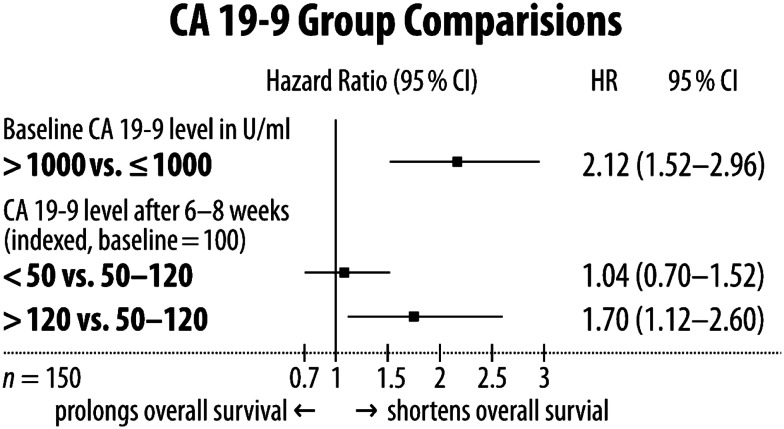
**Forest plot of interfering CA 19-9 serum level characteristics**.

CA 19-9 serum level response after 6–8 weeks of treatment was as follows: patients with decreased CA 19-9 serum level (<50%) had an mOS of 11.1 months (95% CI: 8.85–13.36), patients with stabilization of CA 19-9 serum level (ranging from a decrease of 50% to an increase of 20%) had an mOS of 11.6 months (95% CI: 8.43–14.77), and patients with increased CA 19-9 serum level (>20%) an mOS of 8.1 months (95% CI: 6.56–9.64) (Figures 2 and 3). Patients older than the median age of 64 years did not have poorer mOS [10.8 months (95% CI: 9.87–11.73)] than patients of the median age of 64 years or younger at time of diagnosis [9.3 months (95% CI: 6.94–11.66), log-rank *p* = 0.819; HR: 1.04 (95% CI: 0.75–1.44)].

## Discussion

This was a non-intervention study, so the retrospective method of this investigation was appropriate. Former reports about baseline CA 19-9 serum levels playing an important role in prediction of overall survival are supported by our findings. Patients with lower baseline CA 19-9 serum level (≤1,000 U/ml) had significantly better mOS than patients with a higher CA 19-9 serum level (>1,000 U/ml), indicating more aggressive cancer characteristics or higher cancer burden and thus a higher mortality risk for this patient group [HR 2.12 (95% CI: 1.52–2.96)] (Figure 1). The CA 19-9 serum level characteristic does not seem to have interaction with patients age, because there was no difference in mOS for patients grouped according to a median age of 64 years [HR: 1.04 (95% CI: 0.75–1.44)], indicating that age does not play a significant role in predicting survival.

The main focus of our work was the investigation of changes of serum level CA 19-9 after treatment initiation and its impact on overall survival, similar to other research groups (Ballehaninna and Chamberlain, 2011), except that the other researchers separated the patients only into two groups: response or no response of CA 19-9 serum level. In this studies, serum marker response means a decrease of between 20 and 89% of CA 19-9 serum level (Ballehaninna and Chamberlain, 2011). Results were published as a positive correlation between response and overall survival. We tried to find out whether patients with nearly stabilization of the CA 19-9 serum level had survival disadvantages compared to those patients with high response of CA 19-9 serum level. Thus our analysis was conducted with three different types of CA 19-9 serum level response: in the first group there was a decrease of more than 50%, in the second group with stabilization the response ranged from a decrease of 50% to an increase of 20%, and in the third group there was an increase of more than 20%. Our findings showed that patients with a high CA 19-9 serum level response did not have higher overall survival than patients with stabilized CA 19-9 serum level [HR 1.04 (95% CI: 0.7–1.52)]. Patients who are CA 19-9 serum levels increased by more than 20% had significantly shortened overall survival in comparison to patients with stabilized CA 19-9 serum level [1.7 (95% CI: 1.12–2.6)] (Figures 2 and 3). This implies that an increase in CA 19-9 serum levels of more than 20% after 6–8 weeks of treatment is indicative of an early treatment failure requiring a different antineoplastic regimen if available. In contrast, patients with greatly decreased CA 19-9 serum levels or stabilization might benefit from continuing the regimen. This is remarkable because few research groups assumed that all patients with no CA 19-9 serum level response (e.g., less than 20% decrease) should change to second-line treatment because of resistance to first-line therapy.

The correct timepoint for predictive CA 19-9 serum level measurement has often been discussed. We chose the point between week 6 and 8 of treatment because of the inconsistent amount of this serum marker in the first 4–6 weeks after treatment begin. Thus – similar to other research groups (Vormittag et al., 2009) – we observed elevated CA 19-9 serum levels within the first weeks of treatment in patients with further decline of this biomarker in our own clinical experience.

## Conclusion

Our findings support the importance of monitoring treatment response with carbohydrate antigen 19-9 as a surrogate marker. In summary we advise that patients with decreased and stabilized CA 19-9 serum levels after 6–8 weeks of treatment continue the same first-line treatment regimen, whereas patients with an increase in CA 19-9 serum level of more than 20% should switch to second-line treatment depending on current performance status.

## Authors Contribution

Uwe Pelzer, Andreas Hilbig, and Hanno Riess were responsible for the concept and design of the study and the writing of the manuscript. Data analysis and interpretation was done by Uwe Pelzer, Andreas Hilbig, Marianne Sinn, Jens Stieler, Marcus Bahra, Hanno Riess. Hanno Riess and Bernd Dörken provided staff and facilities for the investigation. All authors were involved in the provision of patients and the collection and collation of data. All authors reviewed the manuscript and gave their approval.

## Conflict of Interest Statement

The authors declare that the research was conducted in the absence of any commercial or financial relationships that could be construed as a potential conflict of interest.
